# Functional genetic variants within the SIRT2 gene promoter in acute myocardial infarction

**DOI:** 10.1371/journal.pone.0176245

**Published:** 2017-04-26

**Authors:** Wentao Yang, Feng Gao, Pei Zhang, Shuchao Pang, Yinghua Cui, Lixin Liu, Guanghe Wei, Bo Yan

**Affiliations:** 1 Department of Medicine, Shandong University School of Medicine, Jinan, Shandong, China; 2 College of Clinical Medicine, Xinxiang Medical University, Xinxiang, Henan, China; 3 Shandong Provincial Key Laboratory of Cardiac Disease Diagnosis and Treatment, Affiliated Hospital of Jining Medical University, Jining Medical University, Jining, Shandong, China; 4 Division of Cardiology, Affiliated Hospital of Jining Medical University, Jining Medical University, Jining, Shandong, China; 5 Shandong Provincial Sino-US Cooperation Research Center for Translational Medicine, Affiliated Hospital of Jining Medical University, Jining Medical University, Jining, Shandong, China; University of Colorado Denver, UNITED STATES

## Abstract

Coronary artery disease (CAD), including acute myocardial infarction (AMI) is the complication of atherosclerosis. Recently, genome-wide association studies have identified a large number of CAD-related genetic variants. However, only 10% of CAD cases could be explained. Low frequent and rare genetic variants have been recently proposed to be main causes for CAD. SIRT2 is a member of sirtuin family, NAD(+)-dependent class III deacetylases. SIRT2 is involved in genomic stability, metabolism, inflammation, oxidative stress and autophagy, as well as in platelet function. Thus, we hypothesized that genetic variants in SIRT2 gene may contribute to AMI. In this study, SIRT2 gene promoter was analyzed in large cohorts of AMI patients (n = 375) and ethnic-matched controls (n = 377). Three novel heterozygous DSVs (g.38900888_91delTAAA, g.38900270A>G and g.38899853C>T) were identified in three AMI patients, but in none of controls. These DSVs significantly altered the transcriptional activity of the SIRT2 gene promoter (P<0.05) in both HEK-293 and H9c2 cells. Five novel heterozygous DSVS (g.38900562C>T, g.38900413A>C, g.38900030G>A, g.38899925A>C and g.38899852C>T) were only found in controls, which did not significantly affected SIRT2 gene promoter activity (P>0.05). In addition, four novel heterozygous DSVs and five SNPs were found in both AMI patients and control with similar frequencies (P>0.05), two SNPs of which were examined and did not affect SIRT2 gene promoter activity (P>0.05). Taken together, the DSVs identified in AMI patients may change SIRT2 level by affecting the transcriptional activity of SIRT2 gene promoter, contributing to the AMI development as a rare risk factor.

## Introduction

Coronary artery disease (CAD) is a common complex disease, including acute myocardial infarction (AMI). The main cause for CAD is atherosclerosis, an inflammatory and metabolic disease. The known risk factors for atherosclerosis and CAD include aging, hypertension, smoking, obesity and diabetes, hyperlipidemia and inflammation. Though genome-wide association studies have identified a great number of genetic loci associated to CAD, these genetic loci collectively explain <10% of CAD cases [1–3]. To date, genetic causes and underlying molecular mechanisms for CAD remain largely unclear. It has been proposed that low frequency and rare genetic variants with large effects account for the missing heritability for human common diseases, including cardiovascular disease [4]. Emerging data suggest that epigenetic factors also contribute to the development of cardiovascular disease [5].

Sirtuins are NAD(+)-dependent class III deacetylases involved in the regulation of cell biological processes, including cellular stress, differentiation, genomic stability, inflammation and metabolism. Human studies and animal experiments have implicated sirtuins in age-related diseases, such as cancer, diabetes, cardiovascular and neurodegenerative diseases [6–8]. In mammals, there are seven sirtuins (SIRT1-7) with diversity in subcellular localization, enzyme activity and function. SIRT2 is localized in both the cytoplasm and nucleus. SIRT2 preferentially deacetylates tubulin and histone H4 and has been involved in multiple cell processes including growth, differentiation, and energy metabolism [9–11]. During the cell cycle, SIRT2 controls mitotic exit, regulates checkpoint pathways and replication stress response, and maintains genome stability [12–15]. In maintaining metabolic homeostasis, SIRT2 has different functions in adipogenesis, fatty acid oxidation, gluconeogenesis, insulin sensitivity and lipid synthesis [16]. SIRT2 is also required in inflammatory process and in response to oxidative stress [17–19]. Recent study indicates that SIRT2 maintains mitochondrial biology and facilitates cell survival by regulating autophagy and mitophagy [20,21]. In human cells, SIRT2 knockdown increases basal autophagy and prevents postslippage death by prolonging chronic mitotic arrest [22,23]. In addition, SIRT2 is expressed in enucleate platelets and plays a central role in platelet function [24,25]. Dysregulated SIRT2 activity has been associated with aging, cancer, metabolic disorders and neurodegeneration [16,26–28].

Accumulating evidence suggest that sirtuins provide protective effects in cardiovascular diseases, mainly SIRT1, SIRT3 and SIRT6 [28,29]. The roles of SIRT2 in the cardiovascular system have recently been studied and reported. In cardiosurgical patients undergoing remote ischemic preconditioning, SIRT2 gene is down-regulated in the cardiac tissue [30]. In human umbilical vein endothelial cells under oxidative stress, SIRT2 regulates the expression of genes involved in cytoskeletal organization, cell contraction and migration, and cell viability [31]. In H9c2 cells and rat cardiomyocytes, short-term calorie restriction activates SIRT2 gene expression [32]. In model animals, SIRT2 regulates microtubule stabilization in diabetic cardiomyopathy [33]. Knockdown or inhibition of SIRT2 enhances biological stress-tolerance in H9c2 cells [34]. SIRT2 gene expression in aorta is significantly reduced in aging mice [35]. In addition, SIRT2 mediates hypertension-induced vascular remodeling [36]. Collectively, these data suggest that SIRT2 plays important roles in the cardiovascular system and may contribute to cardiovascular diseases.

Dysregulated gene expression have been implicated in many human diseases [37]. In previous studies, we have genetically and functionally investigated the members of sirtuin family in AMI patients, including SIRT1, SIRT3 and SIRT6. A number of functional DNA sequence variants (DSVs) within their promoters have been identified and linked to AMI [38–40]. Since SIRT2 has diverse functions in genomic stability, inflammation, metabolism and autophagy, as well as in cardiovascular system, we speculated that SIRT2 may contribute to the CAD development. In this study, the promoter region of the SIRT2 gene were studied in large cohorts of AMI patients and healthy controls.

## Materials and methods

### AMI patients and healthy controls

All AMI patients (n = 375, male 281, female 94, age range from 31 to 85 years, median age 61.00 years) were recruited from April, 2012 to July, 2014, from Cardiac Care Unit, Division of Cardiology, Affiliated Hospital of Jining Medical University, Jining Medical University, Jining, Shandong, China. All AMI patients were diagnosed based on clinical symptoms, electrocardiograph changes (ST-segment elevation or depression), typical rise of biochemical markers of myocardial necrosis (troponin or creatine kinase-MB), or coronary angioplasty. Ethnic-matched healthy controls (n = 377, male 193, female 184, age range from 21 to 84 years, median age 51.00 years) were recruited from the same hospital during the same period. The controls with familial history of CAD were excluded from this study. This study was carried out according to the principles of the Declaration of Helsinki and was approved by the Human Ethic Committee of Affiliated Hospital of Jining Medical University. Written informed consents were obtained from all participants.

### Direct DNA sequencing

Leukocytes were isolated from vein blood and genomic DNAs were extracted. SIRT2 gene promoter region (1446bp, from -1292 bp to +154bp to the transcription start site) was directly sequenced. Two overlapped DNA fragments, 764bp (-1292bp~-521bp) and 678bp (-598bp ~ +154bp), were generated by PCR. PCR primers were designed based on genomic sequence of the human SIRT2 gene (NCBI, NC_000019.10) (Table 1). PCR products were bi-directionally sequenced with Applied Biosystems 3500XL genetic analyzer. The DNA sequences were then aligned and compared with the wild type SIRT2 gene promoter.

**Table 1:** PCR primers for the SIRT2 gene promoter.

Primers	Sequences	Location	Position	Products
Sequencing				
SIRT2-F1	5′-GGCATACAGCAGTAAACACAAC-3′	38901154	-1292	772bp
SIRT2-R1	5′-CTAGCTATGATCCTAACCCAAG-3'	38900383	-521	
SIRT2-F2	5′-ACAATGTGGATTCCAGGAGC-3'	38900460	-598	752bp
SIRT2-R2	5′-TTTGGTACAACACCCAGAGC-3'	38899709	+154	
Functioning				
SIRT2-F	5′-(KpnI)-GGCATACAGCAGTAAACACAAC-3′	38901154	-1292	1446bp
SIRT2-R	5′-(HindIII)-TTTGGTACAACACCCAGAGC-3′	38899709	+154	

PCR primers are designed based on the genomic DNA sequence of the SIRT2 gene (NC_000019.10). The transcription start site (TSS) is at the position of 38899862 (+1).

### Functional analysis with dual-luciferase reporter assay

Wild type and variant SIRT2 gene promoters were subcloned into luciferase reporter vector (pGL3-basic) to construct expression vectors. After transfected into cultured cells, dual-luciferase activities were examined. Briefly, DNA fragments of wild type and variant SIRT2 gene promoters (1446bp, from -1292bp to +154bp to the transcription start site) were generated by PCR and inserted into the KpnI and Hind III sites of pGL3-basic to generate expression vectors. The PCR primers with KpnI or HindIII sites were shown in Table 1. Designated expression vectors were transiently transfected into human embryonic kidney cells (HEK-293) or rat cardiomyocyte line cells (H9c2). Forty-eight hours post-transfection, the cells were collected and luciferases activities were measured using dual-luciferase reporter assay system on a Promega Glomax 20/20 luminometer. Vector pRL-TK expressing renilla luciferase was used as an internal control for transfection. Empty vector pGL3-basic was used as a negative control. The transcriptional activities of the SIRT2 gene promoters were represented as ratios of luciferase activities over renilla luciferase activities. Transcriptional acitivity of the wild type SIRT2 gene promoter was designed as 100%. All the experiments were repeated three times independently, in triplicate.

### Statistical analysis

The quantitative data, including reporter gene expression levels, were represented as mean ± SEM and compared by a standard Student's t-test. The frequencies of hypertension, smoking, type 2 diabetes and DSVs in AMI patients and controls were analyzed and compared with Chi-square test (SPSS v13.0). P<0.05 was considered statistically significant.

## Results

### The DSVs identified in AMI patients and controls

In this study, the prevalence of hypertension was similar in both AMI (28.00%, 105/375) and control groups (31.83%, 120/377) (P>0.05). The prevalence of type 2 diabetes in AMI group (18.93%, 71/375) was significantly higher than that in control group (5.04%, 19/377) (P<0.01). The prevalence of smoking in AMI group (43.60%, 201/375) was also significantly higher than that in control group (7.16%, 27/377) (P<0.01).

**Fig 1:**
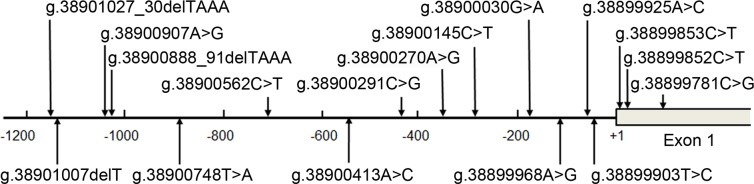
Locations of the DSVs in the SIRT2 gene promoter in AMI patients and controls. The numbers represents the genomic DNA sequences of the human SIRT2 gene (Genebank accession number NC_000019.10). The transcription start site is at the position of 38899862 in the first exon.

A total of 17 DSVs, including 5 single-nucleotide polymorphisms (SNP), were identified in this study. Locations and frequencies of the DSVs were depicted in Fig 1 and summarized in Table 2. Two novel heterozygous DSVs (g.38900270A>G and g.38899853C>T) and one heterozygous deletion DSV (g.38900888_91delTAAA), were identified in three AMI patients, but in none of controls. Clinically, the DSV (g.38900270A>G) was found in a 64-year-old female patient, who had type 2 diabetes, but had no hypertension. The DSV (g.38899853C>T) was found in an 84-year-old female patient, who had no hypertension and type 2 diabetes. The DSV (g.38900888_91delTAAA) was found in a 49-year-old male patient, who had no hypertension and type 2 diabetes. All the three patients were non-smokers. The levels of biochemical parameters in the blood, including triglyceride, total cholesterol, high density lipoprotein cholesterol and high density lipoprotein cholesterol, were all within normal physiological ranges in these patients.

**Table 2:** DSVs within the SIRT2 gene promoters in AMI patients and controls.

DSVs	Genotypes	Location^1^	Controls	AMI	P
g.38901027_30delTAAA	TAAA/-	-1168bp	1	1	1.000
g.38901007delT (rs10713585)	T/T	-1145bp	0	2	0.258
	T/-		21	15	
	-/-		356	358	
g.38900907A>G (rs4803006)	AA	-1045bp	376	373	0.624
	GG		1	2	
g.38900888_91delTAAA	TAAA/-	-1029bp	0	1	-
g.38900748T>A	TA	-878bp	3	3	1.000
g.38900562C>T	CT	-700bp	1	0	-
g.38900413A>C	AC	-551bp	1	0	-
g.38900291C>G (rs2053071)	CC	-429bp	72	78	0.353
	CG		178	189	
	GG		127	108	
g.38900270A>G	AG	-348bp	0	1	-
g.38900145C>T (rs116900177)	CT	-283bp	18	19	0.868
g.38900030G>A	GA	-168bp	1	0	-
g.38899968A>G (rs112492606)	AG	-106bp	1	1	1.000
g.38899925A>C	AC	-63bp	1	0	-
g.38899903T>C	TC	-41bp	1	1	1.000
g.38899853C>T	CT	+10bp	0	1	-
g.38899852C>T	CT	+11bp	1	0	-
g.38899781C>G	CG	+82bp	1	2	0.624

^1^, DSVs are located upstream (-) to the transcription start site of SIRT2 gene at 38899862 of NC_000019.10.

**Fig 2:**
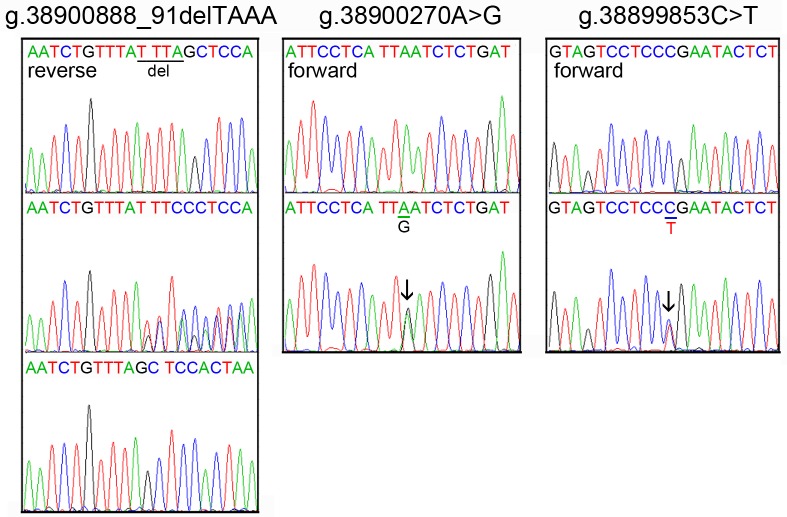
Sequencing chromatograms of the novel DSVs in AMI patients. Sequence orientations of the DSVs are marked. For DSVs g.38900270A>G and g.38899853C>T, top panels show wild type and bottom panels heterozygous DNA sequences, which are marked with arrows. For the deletion DSV g.38900888_91delTAAA, top panel shows wild type, middle panel heterozygous and bottom panel cloning DNA sequence. The deletion is underlined and labeled.

**Fig 3:**
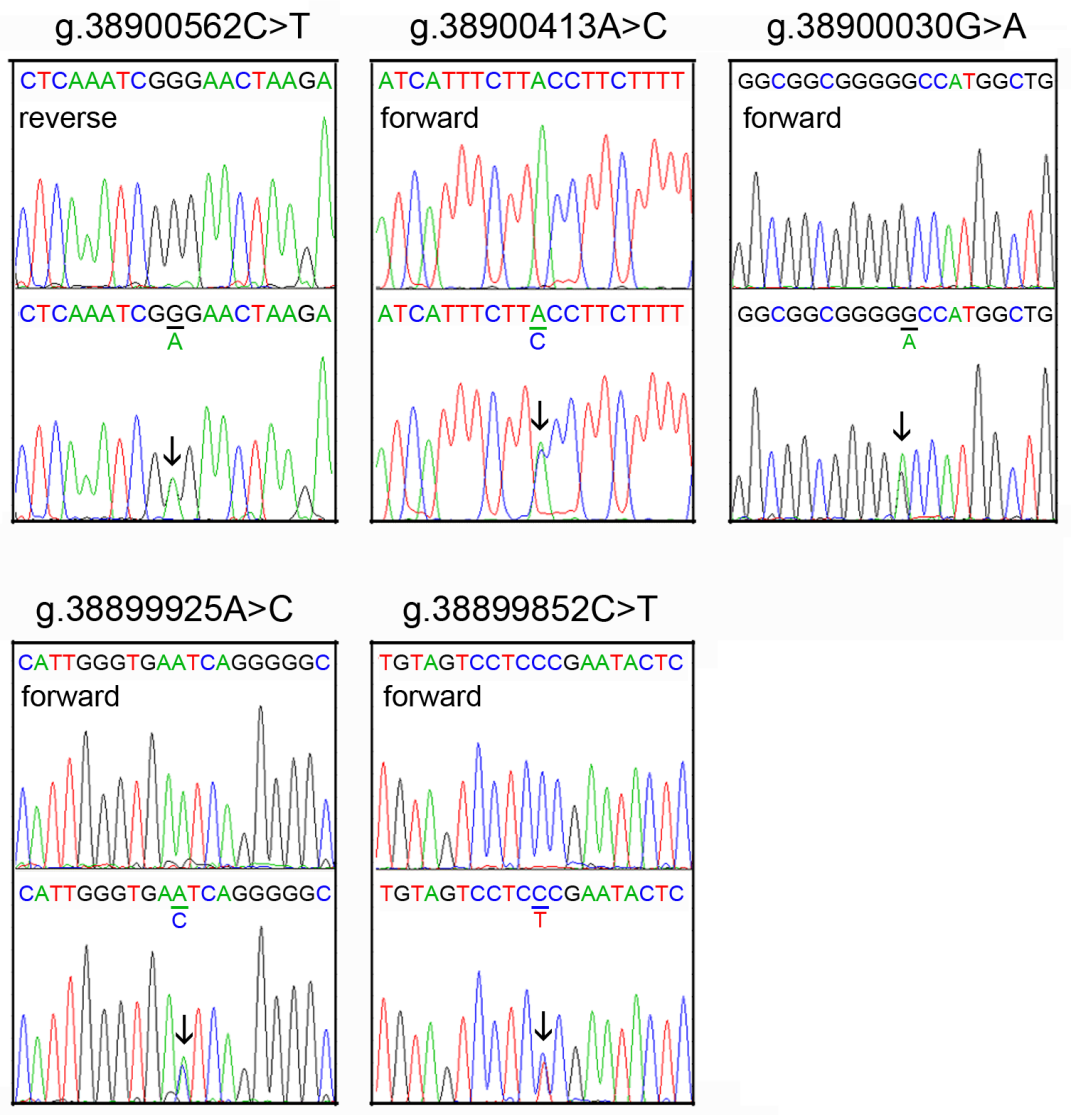
Sequencing chromatograms of the novel DSVs only identified in controls. For all the novel heterozygous DSVs (g.38900562C>T, g.38900413A>C, g.38900030G>A, g.38899925A>C and g.38899852C>T), top panels show wild type and bottom panels heterozygous DNA sequences, which are marked with arrows.

**Fig 4:**
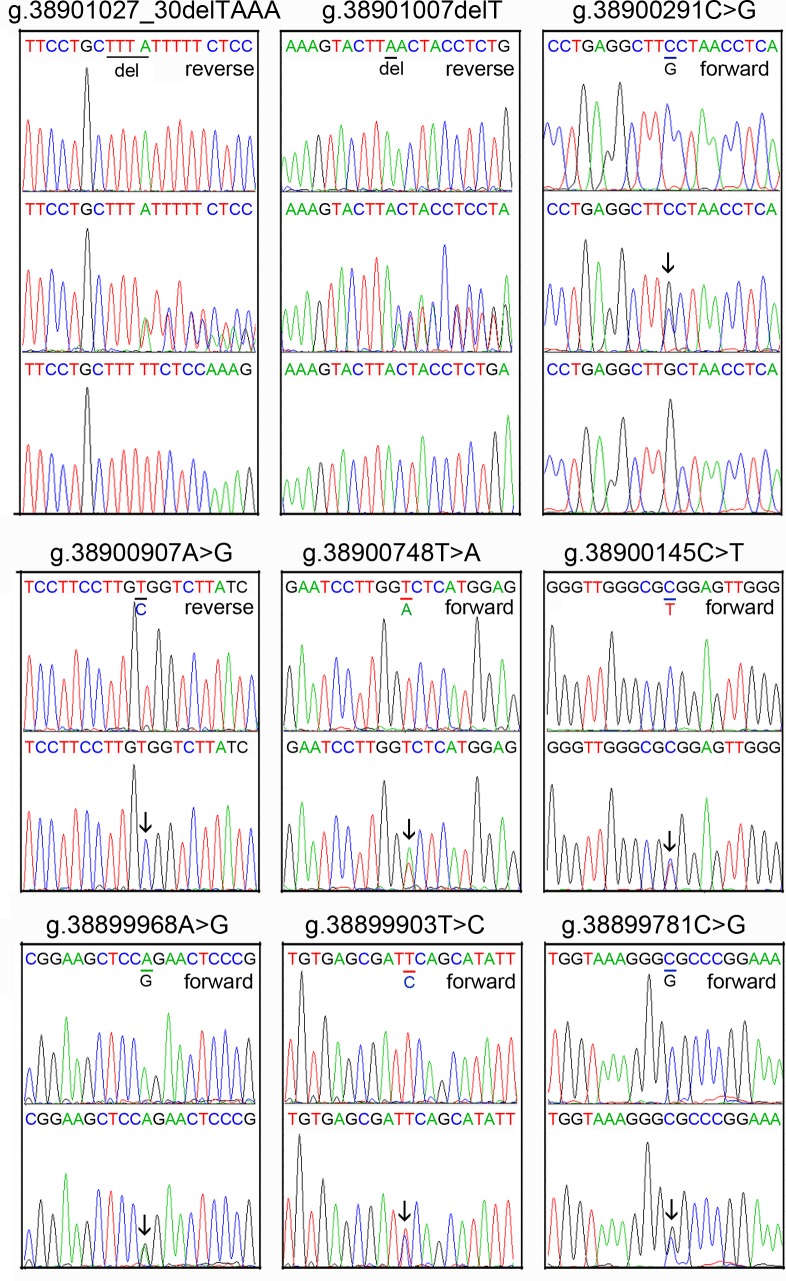
Sequencing chromatograms of the DSVs in both AMI patients and controls. The nine DSVs include one novel deletion DSV, five SNPs and three novel heterozygous DSVs. For deletion DSV (g.38901027_30delTAAA) and deletion SNP [g.38901007delT (rs10713585)], top panel shows wild type, middle panel heterozygous and bottom panel cloning DNA sequence. The deletions are underlined and labeled. For SNP [g.38900291C>G (rs2053071)], top panel shows wild type, middle panel heterozygous and bottom panel homozygous DNA sequence. For SNP g.38900907A>G (rs4803006), top panel shows wild type and bottom panel homozygous DNA sequence. For other novel heterozygous DSVs (g.38900748T>A, g.38899903T>C and g.38899781C>G), and SNPs [g.38900145C>T (rs116900177) and g.38899968A>G (rs112492606)], top panels show wild type and bottom panels heterozygous DNA sequences, which are marked with arrows.

The DNA sequencing chromatograms of these novel DSVs were shown in Fig 2. Five novel heterozygous DSVS (g.38900562C>T, g.38900413A>C, g.38900030G>A, g.38899925A>C and g.38899852C>T) were only found in healthy controls, DNA sequencing chromatograms of which were shown in Fig 3. In addition, three novel heterozygous DSVs (g.38900748T>A, g.38899903T>C and g.38899781C>G), one deletion DSV (g.38901027_30delTAAA) and five SNPs [g.38901007delT (rs10713585), g.38900907A>G (rs4803006), g.38900291C>G (rs2053071), g.38900145C>T (rs116900177) and g.38899968A>C (rs112492606)] were found in both AMI patients and controls with similar frequencies (P>0.05) (Fig 4).

### Putative binding sites for transcription factors affected by DSVs

To determine whether DSVS affect putative biding sites for transcription factors, the SIRT2 gene promoter was analyzed with JASPAR program (http://jaspar.genereg.net/). The DSVs identified in AMI patients may abolish, create or modify the putative binding sites for transcription factors. The DSV g.38900888_91delTAAA may abolish binding sites for forkhead box (FOX) transcription factors, including FOXD2, FOXL1, FOXO4, FOXO6, FOXP2 and FOXP3. The DSV g.38900270A>G may abolish GS homeo Box Protein 1 (GSX1), homeo Box factor B3 (HOXB3), HOX-related factor PDX1, NK-related homeodomain factors (BSX, NKX6-1, NKX6-2 and NOTO), orthodenticle homeobox factor 2 (OTX2) and PU domain factor POU6F2. The DSV g.38899853C>T may abolish heat shock factor 4 (HSF4), THAP-related zinc finger factor THAP1 and myeloid zinc finger gene 1 (MZF1).

### Functional analysis of the DSVs by dual-luciferase reporter assay

Wild type and variant SIRT2 gene promoters were cloned into luciferase reporter vector (pGL3-basic) to generate expression vectors, including empty pGL3-basic (negative control), pGL3-WT (wild type SIRT2 gene promoter), pGL3-38900907G, pGL3-38900888_91del, pGL3-38900562T, pGL3-38900413C, pGL3-38900291G, pGL3-38900270G, pGL3-38900030A, pGL3-38899925C, pGL3-38899903C, pGL3-38899853T, pGL3-38899852T and pGL3-38899781G. After transfected into cultured cell lines, human embryonic-kidney cells (HEK-293) and rat cardiomyocyte cells (H9c2), the cells were collected and dual-luciferase activities were assayed and relative transcriptional activities of the SIRT2 gene promoters were calculated. Transcriptional activity of the wild type SIRT2 gene promoter was set as 100%.

**Fig 5:**
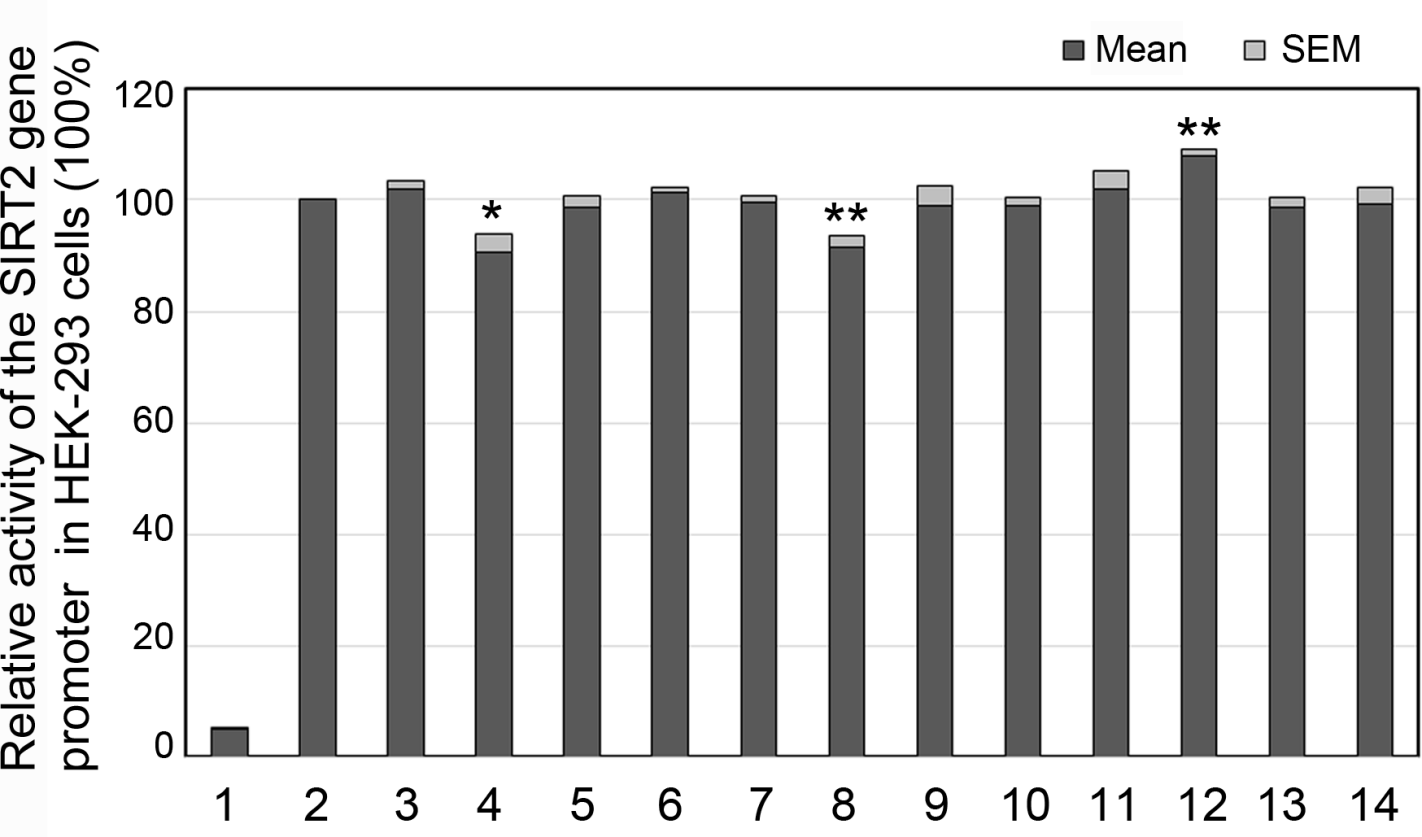
Relative activities of wild type and variant SIRT2 gene promoters in HEK-293 cells. Wild type and variant SIRT2 gene promoters were cloned into reporter gene vector pGL3 and transfected into HEK-293 cells. The transfected cells were collected and dual-luciferase activities were assayed. Empty vector pGL3-basic is used as a negative control. Transcriptional acitivity of the wild type SIRT2gene promoter was designed as 100%. Relative activities of SIRT2 gene promoters were calculated. Lanes 1, pGL3-basic; 2, pGL3-WT; 3, pGL3-38900907G; 4, pGL3-38900888_91del; 5, pGL3-38900562T; 6, pGL3-38900413C; 7, pGL3-38900291G; 8, pGL3-38900270G; 9, pGL3-38900030A; 10, pGL3-38899925C; 11, pGL3-38899903C; 12, pGL3-38899853T; 13, pGL3-38899852T; 14, pGL3-38899781G. WT, wild type. *, P<0.05; **, P<0.01.

In HEK-293 cells, the DSVs (g.38900888_91delTAAA and g.38900270A>G) that were identified only in AMI patients significantly decreased activity of the SIRT2 gene promoter (90.73% ± 3.28%, P<0.05 and 91.60% ± 1.86%, P<0.01, respectively). The DSV (g.38899853C>T) only identified in an AMI patient significantly increased activity of the SIRT2 gene promoter (107.90% ± 1.27%, P<0.01). The DSVs (g.38900562C>T, g.38900413A>C, g.38900030G>A, g.38899925A>C and g.38899852C>T) that were found only in controls did not significantly alter activity of the SIRT2 gene promoter (P>0.05). As expected, the SNPs [g.38900907A>G (rs4803006) and g.38900291C>G (rs2053071)] and the DSVs (g.38899903T>C and g.38899781C>G) that were found in both AMI patients and controls did not also alter activity of the SIRT2 gene promoter (P>0.05) (Fig 5).

**Fig 6:**
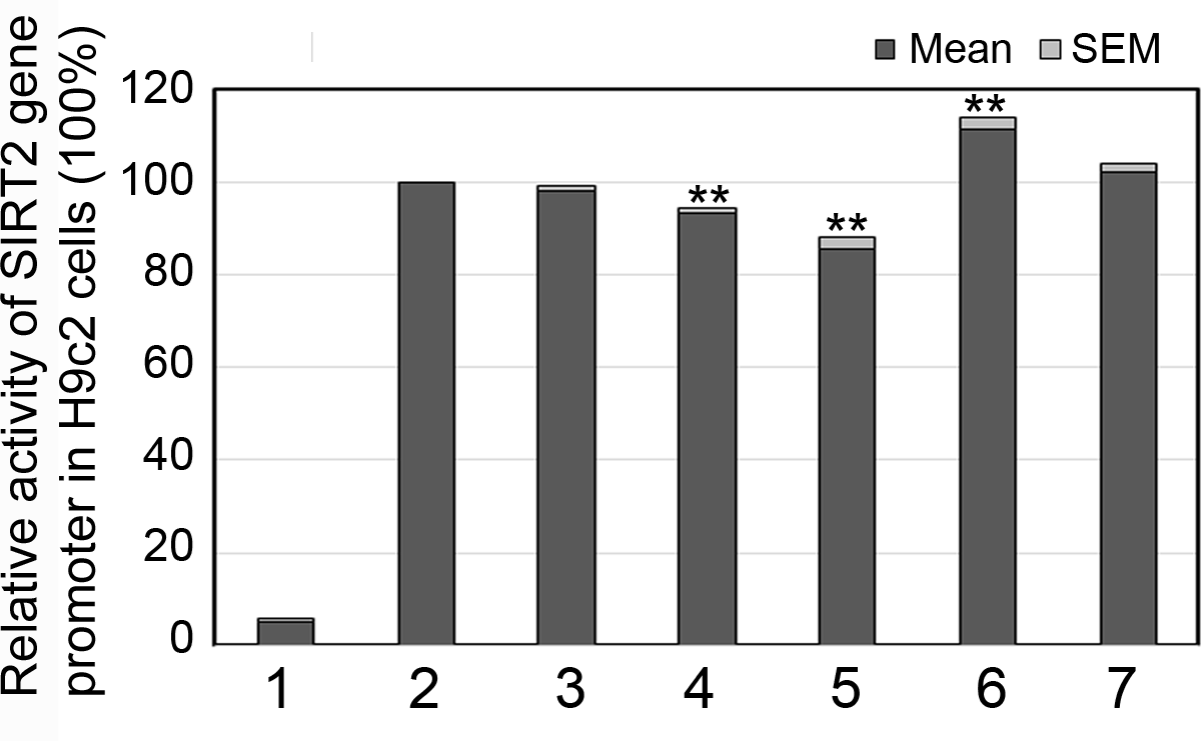
Relative activities of wild type and variant SIRT2 gene promoters in H9c2 cells. Expression constructs for wild type and variant SIRT2 gene promoters were transfected into H9c2 cells and dual-luciferase activities were measured. Transcriptional acitivity of the wild type SIRT2 gene promoter was designed as 100%. Relative activities of SIRT2 gene promoters were calculated. Lanes 1, pGL3-basic; 2, pGL3-WT; 3, pGL3-38900907A>G; 4, pGL3-38900888_91del; 5, pGL3-38900270G; 6, pGL3-38899853T; 7, pGL3-38900291G. **, P<0.01.

To further investigate the tissue specificity of the DSVs in cardiomyocytes, we examined the transcriptional activity of the variant DSVs found in AMI patients (g.38900888_91delTAAA, g.38900270A>G and g.38899853C>T) in H9c2 cells. The SNPs [g.38900907A>G (rs4803006) and g.38900291C>G (rs2053071)] found in both AMI patients and controls were also tested as internal controls. Consistent to the above transfection results in HEK-293 cells, the DSVs (g.38900888_91delTAAA and g.38900270A>G) significantly decreased the activity of the SIRT2 gene promoter (93.23% ± 1.11%, P<0.01 and 85.66% ± 2.43%, P<0.01, respectively) and the DSV (g.38899853C>T) significantly increased the activity of the SIRT2 gene promoter (111.30% ± 2.67%, P<0.01). Similarly, the SNPs [g.38900907A>G (rs4803006) and g.38900291C>G (rs2053071)] did not significantly alter the activity of the SIRT2 gene promoter (P>0.05) (Fig 6). Taken together, the DSVs identified in AMI patients altered the activity of the SIRT2 gene promoter in both HEK-293 cells and H9c2 cells, suggesting their non-tissue specific effects.

## Discussion

Genetic variants in SIRT2 gene have been associated with human traits and diseases. The SIRT2 gene SNP (rs45592833G/T), which is located in the 3'-untranslated regions (3'UTR), is significantly associated with human longevity [41]. SIRT2 gene SNPs, rs2241703 in the 3’ untranslated region and rs11879029 in an intron, have been reported to be associated with height in a Japanese population [42]. The intronic SNP (rs10410544) in the SIRT2 gene significantly increases risk of Alzheimer's disease [43]. In this study, three novel heterozygous DSVs (g.38900888_91delTAAA, g.38900270A>G and g.38899853C>T) were identified in three AMI patients, but in none of controls. These DSVs significantly altered the transcriptional activity of the SIRT2 gene promoter (P<0.05) in both HEK-293 and H9c2 cells. Therefore, these SIRT2 gene promoter DSVs may change SIRT2 levels, contributing to the AMI development as a risk factor.

The human SIRT2 gene has been localized to chromosome 19q13.1, which has 16 exons and spans a region of 20,960 bp. SIRT2 gene is widely expressed in fetal and adult tissues with higher expression in heart, brain, and skeletal muscle, and lower expression in placenta and lung [44,45]. The SIRT2 gene promoter is a TATA- and CCAAT-box less promoter, containing a 670bp CpG island and a number of NF-κB and GATA transcription factor binding sites [46]. In human cells, SIRT2 gene is directly regulated by P53 [47]. In addition, SIRT2 gene is a direct target of microRNA-7 [48]. In this study, three functioning DSVs in SIRT2 gene promoter were identified. Further investigation to find transcription factors binding to these DSVs will provided a piece of new information for characterizing the human SIRT2 gene promoter.

Altered expression of SIRT2 gene has been observed in aging process and human diseases. SIRT2 level in human peripheral blood mononuclear cells decreases with aging process [49]. SIRT2 gene expression in human mononuclear cells is upregulated by caloric restriction [50]. Low levels of SIRT2 are detected in visceral adipose tissue from human obese subjects [51]. In human endothelial cells, SIRT2 gene is downregulated with increasing passage, further confirming the above SIRT2 level changes in vivo [52]. SIRT2 levels have been associated with the pathogenesis of Parkinson’s disease [48,53]. In diverse types of cancers, SIRT2 gene expression is downregulated or upregulated [54–57]. In this study, genetic variants in SIRT2 gene promoter may change SIRT2 levels in AMI patients. Therefore, the human SIRT2 gene expression may be manipulated with genetic approaches or pharmatheutical agents for the therapeutic purposes.

SIRT2 deacetylates and interacts with proteins functioning in different cellular processes, including tubulin, histones and transcription factors. In the regulation of cell cycle and genomic stability, SIRT2 is the deacetylase for alpha-tubulin in controlling microtubule stability, CDH1 and CDC20 in regulating APC activity, histone H3K56 in response to DNA damage, H4K16 in modulating H4K20 methylation levels, ankyrin repeat and LEM domain-containing protein 2 (ANKLE2) in nuclear envelope reassembly, CDK9 activity in response to replication stress and the core mitotic checkpoint protein BubR1 [9,10,14,58–63]. SIRT2 is colocalized and interacts with group IVA cytosolic phospholipase A2 (cPLA2α), promoting G2-to-M transition [64]. In contrast, SIRT2 is post-transcriptionally regulated by cyclin-dependent kinases, which functions in cell cycle progression and cytoskeletal dynamics [65]. Genomic stability is one of hallmarks of aging and has been associated with age-associated diseases [66].

SIRT2 has been involved in cell growth, cell death and development by interacting with a broad range of transcription factors, coregulators and signaling molecules. In human cells, SIRT2 deacetylates P300 and P53 proteins, and interacts with homeobox transcription factor HOXA10 [67–69]. In response to oxidative stress, SIRT2 deacetylates FOXO3a and increases its target gene expression to promote cell death [70]. SIRT2 negatively regulates adipocyte differentiation primarily by deacetylation of FOXO1, a mediator of autophagy [71,72]. FOXO1 is crucial for sustaining cardiomyocyte metabolism and cell survival. Down-regulation of FOXO1 in endothelial tissue could prevent against atherosclerotic plaques [73]. A window of optimal autophagic activity is critical to the maintenance of cardiovascular homeostasis and function [74]. The hyperacetylation of alpha-tubulin, a main substrate of SIRT2, promotes autophagy in cardiomyocytes [75]. SIRT2 colocalizes and interacts with histone deacetylase (HDAC6), another tubulin deacetylase [10]. HDAC6 is involved in protein trafficking and degradation, cell shape and migration, and regulates cardiac contraction and protein aggregation [76]. In human cells, SIRT2 is responsible for the acetylation of p70 ribosomal S6 kinase (S6K1), a major substrate of the mammalian target of rapamycin (mTOR) kinase [77]. SIRT2 regulates platelet function by the acetylation and inhibition of Akt kinase, which is implicated in the control of cellular growth, angiogenesis, apoptosis, autophagy, and aging [78].

SIRT2 has been shown to regulate glucose homeostasis, lipid metabolism and inflammation. SIRT2 regulates glucose metabolism by deacetylating M2 isoform of pyruvate kinase (PKM2), glucose-6-phosphate dehydrogenase (G6PD) in the pentose phosphate pathway, glucokinase regulatory protein (GKRP), glycolytic enzyme phosphoglycerate mutase (PGAM) and lactate dehydrogenase A (LDH-A) [19,79–82]. SIRT2 deacetylates and destabilizes ATP citrate lyase (ACLY) in lipid synthesis by regulating production of acetyl coenzyme A [83]. In model animals, SIRT2 regulates sterol biosynthesis by influencing nuclear trafficking of sterol response element binding protein 2 (SREBP-2) [84]. Hif1a is associated with dietary obesity by restricting fatty acid oxidation through repression of SIRT2 [85]. SIRT2 cytoplasmic functions are also involved in intracellular trafficking pathways to maintain cellular homeostasis [86]. In experimental animals, SIRT2 modulates inflammatory response and regulates microvascular inflammation through deacetylation NF-κB p65 [87,88]. SIRT2-mediated H3K18 deacetylation also plays a critical role in bacterial infection [89]. Inflammation is also involved in the initiation and progression of atherosclerosis and its complications, particularly plaque rupture and acute AMI [90].

The protective or detrimental effects of SIRT2 depend on the cell types and stimulations under oxidative stress [19,91]. The opposite effects of SIRT1 and SIRT2 have been reported, suggesting that alterations of SIRT1:SIRT2 expression ratio may be involved in human diseases [53,92]. Increased or decreased SIRT2 levels may contribute to human diseases by disrupting the genomic stability, lipid metabolism, inflammation, autophagy and other signaling pathways. In this study, the genetic variants in SIRT2 gene promoter may downregulate or upregulate SIRT2 gene expression and change SIRT2 levels, contributing to AMI development as a rare risk factor. Precise molecular mechanisms by which the genetic variants in SIRT2 gene promoter affect its gene expression are being carried out in our laboratory.

## Conclusions

In the present study, the SIRT2 gene promoter was genetically and functionally analyzed in AMI patients and healthy controls. The novel DSVs identified in AMI patients significantly altered the transcriptional activity of the SIRT2 gene promoter in cultured cardiomyocytes. Therefore, the SIRT2 gene promoter DSVs may alter transcriptional activity of SIRT2 gene promoter and change SIRT2 level, contributing to AMI development as a rare risk factor. The molecular mechanisms by which the DSVs influence SIRT2 gene expression are being explored in our laboratory. Our findings may provide a genetic basis for translational and therapeutic studies for AMI patients.
